# Molecular Targets and Treatment of Meningioma

**Published:** 2014-04-07

**Authors:** Rickey Miller, Michele L. DeCandio, Yaenette Dixon-Mah, Pierre Giglio, W Alex Vandergrift, Naren L. Banik, Sunil. J. Patel, Abhay K. Varma, Arabinda Das

**Affiliations:** 1 Department of Neurosciences (Divisions of Neurology and Neurosurgery) & MUSC Brain & Spine Tumor Program Medical University of South Carolina, Charleston, SC 29425, USA; 2 Ralph H. Johnson VA Medical Center, Charleston, SC, USA

**Keywords:** Meningioma, Tumorgenesis, Molecular genetics, NF2, Merlin, Proliferation

## Abstract

Meningiomas are by far the most common tumors arising from the meninges. A myriad of aberrant signaling pathways involved with meningioma tumorigenesis, have been discovered. Understanding these disrupted pathways will aid in deciphering the relationship between various genetic changes and their downstream effects on meningioma pathogenesis. An understanding of the genetic and molecular profile of meningioma would provide a valuable first step towards developing more effective treatments for this intracranial tumor. Chromosomes 1, 10, 14, 22, their associated genes, and other potential targets have been linked to meningioma proliferation and progression. It is presumed that through an understanding of these genetic factors, more educated meningioma treatment techniques can be implemented. Future therapies will include combinations of targeted molecular agents including gene therapy, si-RNA mediation, proton therapy, and other approaches as a result of continued progress in the understanding of genetic and biological changes associated with meningiomas. This review provides an overview of the current knowledge of the genetic, signaling and molecular profile of meningioma and possible treatments strategies associated with such profiles.

## Introduction

Menigiomas are the second-most common central nervous system tumor in adults [1–4]. These tumors arise from arachnoid cells of the meninges, the covering layer of the brain. The majority of meningiomas tend to be benign, localized, and non-invasive. However, some meningiomas tend to be more aggressive with tendencies toward invasion of the surrounding brain, high propensity for recurrence, and in rare cases extracranial metastasis [1]. There has been some progress in understanding the molecular genetics of meningiomas, but compared to our knowledge of gliomas, relatively little is known [2–4]. Based on their histopathological characteristics meningiomas are classified as grade I (benign), grade II (atypical), and grade III (malignant/anaplastic) (Table 1). The grade I category is the most prevalent (78–81%), followed by the atypical variety (15–20%), and only 1–4% of meningiomas are anaplastic or malignant [1]. Benign meningioma grows slower, is non-cancerous, and doesn’t meet the criteria to be classified as atypical or anaplastic. Grade I (benign) meningiomas exhibit histologic patterns other than papillary, chordoid, clear cell, or rhabdoid (Table 1) [5]. Although less frequent than benign meningioma, atypical meningiomas grow faster than grade I meningiomas and are characteristically more aggressive. Furthermore, grade II (atypical) meningiomas have 4 to 19 mitoses, present 3 or the following 5 parameters (Macronuclei, spontaneous necrosis, hypercellularity, small cell formation, sheeting architecture), and have clear cell or chordoid cell types (Table 1) [5,6]. Grade III meningiomas are cancerous aggressive and have a differential diagnosis including other malignant neoplasms because histologically they can resemble other tumors (melanomas, carcinomas, and sarcomas). Additionally, anaplastic meningiomas have 20 or more mitoses, anaplasia has been noted (which is similar histology to carcinomas and sarcomas), and have varying cell types (rhabdoid or papillary) (Table 1) [5]. Understanding these histopathological characteristics is how the World Health Organization (WHO) establishes the grading scale for meningiomas (Table 1).

## Genetics

The pathogenesis and molecular genetics of meningioma are not well understood, though a variety of chromosomal, signaling pathway and growth factor alterations have been described [6,7]. Progress in molecular genetics probably represents the most important accomplishment in the comprehensive knowledge of meningioma pathogenesis. Several genes have been identified as targets for mutation or inactivation [8]. Additional chromosomal regions have been found to be commonly deleted or amplified, suggesting the presence of further tumor suppressor genes or proto-oncogenes, respectively, in these regions [9]. Many genetic factors and pathways have been linked to the proliferation, tumorgenesis, progression, and recurrence of meningiomas. Cell cycle dysregulation and telomerase activation have been recognized as important steps in meningioma progression. Telomere dynamics, cell cycle control, and the mechanisms responsible for deoxyribonucleic acid damage control are tightly interwoven [8]. An area that has received relatively little attention thus far is the genetic background of meningioma spread and invasion. The integration of histopathological appearance, complex genetic/genomic data, and outcome will likely result in the identification of clinically distinct meningioma subgroups, which in turn can facilitate the development of targeted therapeutic strategies. Investigating genes involved in the maintenance of genomic integrity might significantly deepen the understanding of meningioma progression.

### Molecular genetics of grade I meningioma (benign)

The integration of histopathological appearance, complex genetic/genomic data, and outcome will likely result in the identification of clinically distinct meningioma subgroups, which in turn can facilitate the development of targeted therapeutic strategies. Investigating genes involved in the maintenance of genomic integrity might significantly deepen the understanding of meningioma progression. Meningiomas can exhibit a number of genetic mutations and aberrations in the human genome. The majority of WHO grade I meningiomas (50%–60%) have been linked to mutations of the NF2 gene on chromosome 22 (location: q12.2) [10–13]. Mutations in this region lead to the condition known as neurofibromatosis type II where benign tumors proliferate throughout the central nervous system (most commonly schwannomas and meningiomas).

The NF2 gene codes for the protein merlin that acts as a tumor suppressor for many different cell types. The NF2 gene product belongs to the 4.1 family of structural proteins that associate integral membrane proteins to the cytoskeleton. It shows strong similarity to the proteins ezrin, radixin, and moesin (ERM proteins) and therefore was named merlin, for moesin, ezrin and radixin-like protein. Different types of meningiomas have shown varying levels of merlin loss, which may explain a change in treatment paradigm for the varying subsets of meningioma. Meningothelial meningiomas tend to express lower levels of merlin loss than fibrous and other forms of meningiomas [14].

Merlin interacts with the intermolecular amino-terminal domain (NTD) and the carboxyl-terminal domain (CTD) through phosphorylation that also controls the binding to its effector proteins. Merlin is localized to the cell membrane at regions that regulate cell-cell contact and motility. Several merlin-interacting proteins have been identified. One class includes cell-surface proteins that bind to FERM-containing proteins, such as CD44 and *β*1-integrin. These molecules involved in cytoskeleton dynamics (*β*II-spectrin, paxillin, actin, and syntenin) represented another class. Lastly, molecules have been identified that may be important for regulation of ion transport, such as the sodium–hydrogen exchange regulatory factor, and endocytosis, such as the hepatocyte growth factor-regulated, tyrosine kinase substrate.

**Figure 1:**
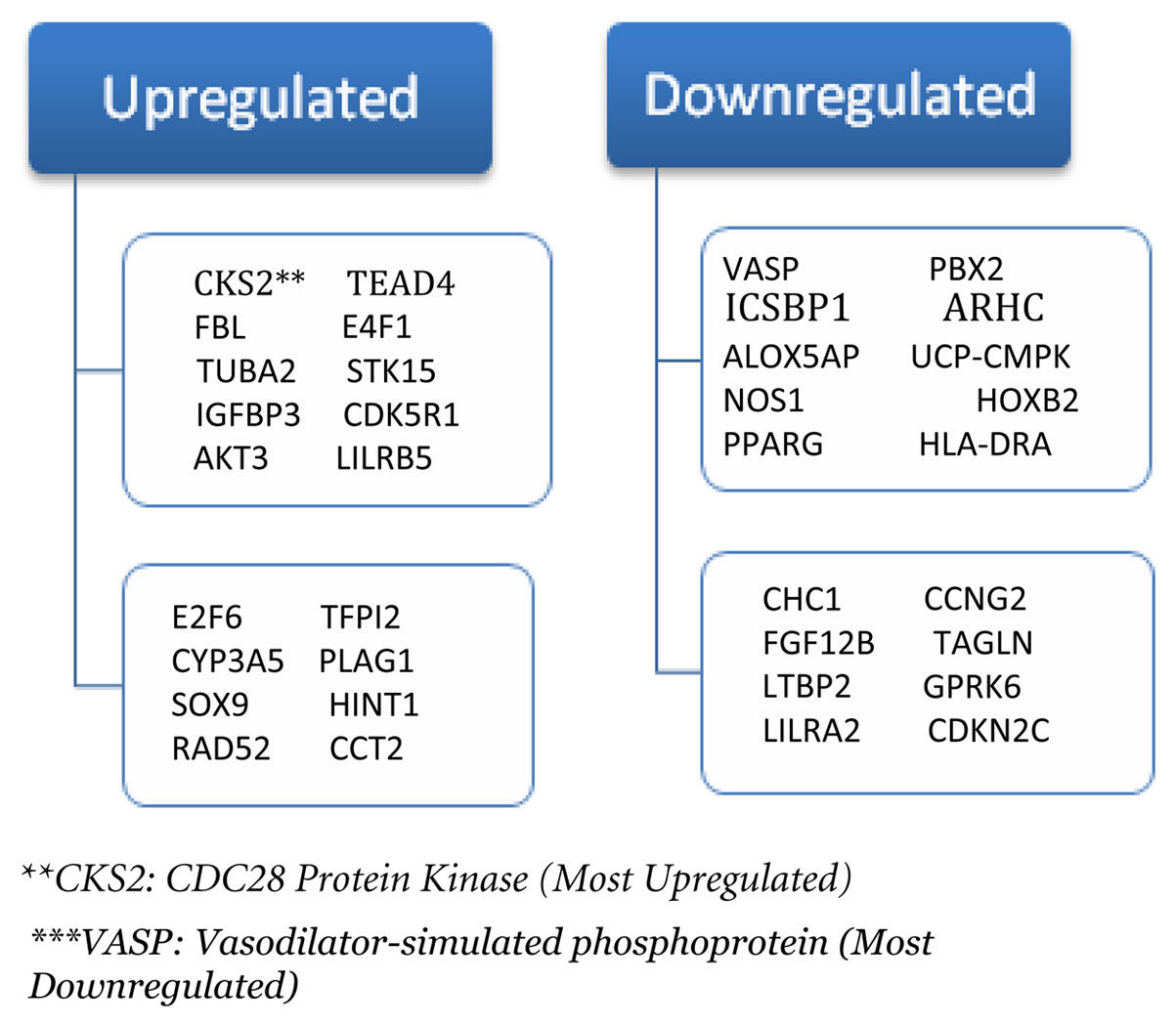
Meningiomaupregulation and downregulation. These are the genes that are upregulated and downregulated by menigiomas in the CNS. The measure to which a gene is upregulated or downregulated is recorded on an ROC curve and that gene is given a value. The higher a gene’s ROC value is the more upregulated or downregulated a gene is. CKS2 has an ROC value of 0.743 which is highest among all upregulated genes while VASP has an ROC value of 0.89 which is highest among all downregulated genes [8].

When the NF2 gene is mutated, merlin production is impaired that leads to an increase of Yes-associated protein (YAP) levels in the CNS that can lead to meningioma proliferation. In primary tumor and meningioma cell lines, YAP overexpression and nuclear localization have been found in cells with merlin loss (NF2 mutation) [7]. Merlin regulates YAP levels, which controls cell proliferation and the unintentional entry into the S-phase of the cell cycle. Besides NF2 mutations with loss of merlin expression, other protein 4.1 family members are also down regulated in meningioma. Loss of protein 4.1B (DAL-1) expression is another possible aberration detected in grade I meningiomas. The loss of both DAL-1 and Merlin has been observed in 50% of benign meningioma [15]. The TSLC-1 gene (tumor suppressor gene for lung cancer-1) produces a protein that interacts with DAL-1; however, in about 30–85% of sporadic meningiomas loss of this gene have been shown [16]. Additionally, epidermal growth factor receptors (EGFR) tend to be overexpressed in benign meningiomas [17], and the platelet-derived growth factor receptor beta (PDGFRB) gene is also upregulated and overexpressed in benign meningioma (Figure 1).

### Molecular genetics of grade II/III meningioma (Atypical/anaplastic; malignant)

Approximately 60% of grade II meningiomas have shown point mutations (mainly deletions) in chromosomes [18]. Furthermore, 75%–90% of grade III meningiomas have similar point mutations in chromosomes [18]. Mutations in the 1p and 14q regions of the chromosome (where tumor suppressor genes are believed to e housed) are associated with a worse prognosis. Chromosome 1 alterations aren’t common in the cell lines of Grade-1 meningiomas but have been noted to contribute to the aggressiveness and overall progression of grade III meningioma. Two main target regions on 1p33–34 and 1p36 are involved. Recent fine mapping narrowed the smallest region of overlapping deletion on 1p33–p34 to 2.8 megabases. The region on 1p36 spans about 8–21 megabases of chromosomal sequence. Several candidate genes on 1p have been screened, including TP73, CDKN2C (encoding p18INK4c), RAD54L, and ALPL [19]. However, none of these genes showed any consistent structural alterations. Table 2 lists a number of genes associated meningiomas as well as their genetic products and functions. Understanding the mutations in the tumor suppressor genes of chromosome-1 is the key to mapping the progression of these tumors and a critical step to containment, control and possible eradication of more aggressive meningiomas.

The PTEN gene is a tumor suppressor gene located near the p23.3 region of chromosome 10 and it produces a protein knownas phosphatidylinositol-3, 4, 5-trisphosphate 3-phosphate [20,21]. It negatively regulates the AKT/PKB pathway that has been linked to the pathogenesis and proliferation of meningiomas and other tumors [22,23]. Even though many tumors lack PTEN functionality, this gene doesn’t contribute to lower grade tumor proliferation but has a role in the proliferation and progression of higher-grade tumors (WHO Grades II and III). Mutations of chromosomes 10 & 14 have been linked to higher-grade meningiomas through deletions. Functional loss of these two chromosomes are associated with expression profiles of some genes mainly overexpression of genes in the Wnt pathway (CTNNB1, CDK5R1, ENC1, CCND1) and insulin-like growth factor (IGF2, IGFBP3, AKT3) [12,24–25]. Furthermore, alterations to the SUFU gene on chromosome 10 (location 10q24) have been linked to meningioma proliferation in grade II and III tumors through heterozygous germline mutations in this gene.

Other genetic non-NF2 aberrations of oncogenes have been observed in skull base and higher-grade meningiomas [12,24–26]. The Glu17Lys mutation of the AKT1 (chromosome 14q32.32) gene was one of the mutations found in tumor cell lines that had no genetic alterations in the NF2 gene. This causes an over active Akt kinase (protein kinase B) which excessively phosphorylates factors of the AKT/PKB pathway when it should be in the “off” position. This leads to an increase in the level of YAP that potentially can suppress the expression of pro-apoptotic genes that activate after cellular DNA damage [12,23–26]. The GADD45A was once thought to be a potential genetic target for meningioma and other tumor pathogenesis; however, no genetic abnormalities were found in meningioma cell lines. The IMP3 gene is another gene of interest because the IMP3 protein has been identified as a biomarker for the aggressiveness of tumors [26]. Mutations in chromosome 9 also has been linked to meningioma proliferation mainly WHO Grade III meningiomas. 9p losses have been found in 5% of benign meningiomas, 18% of atypical meningiomas, and 38% of anaplastic meningiomas [27–29]. Loses of the CDKN2A (p16), p14 (ARF), and CDKN2B (p15) are the main alterations that have been linked to chromosome 9 and are primarily found in anaplastic meningiomas. p15, p14, and p16 are tumor suppressor genes found at 9p21 that play a role in cell apoptosis regulation through modulation (p14) and cell cycle progression through G1 to S phase (p15 and p16) [30]. Figure 1 shows other genes that are upregulated and downregulated by meningioma. Just like in benign meningiomas, grade II/III meningiomas have shown losses in the TSLC-1 gene and its protein, DAL-1, and Merlin. DAL-1 (4.1B protein) and Merlin losses have been observed in 60% of atypical meningiomas and 70% of anaplastic meningiomas [6]. This combination loss of DAL-1 and Merlin may be a precursor for aggressiveness in meningiomas as the grade increase the severity of the meningioma increases along with the absence of Merlin and DAL-1 [31–34]. Other genetic abnormality that leads to higher-grade meningiomas includes: Losses in 6q and 18q and gains in 1q, 9q, 12q, 15q, 17q, and 20q [35,36].

## Genes and growth factors in signaling pathways

**Figure 2:**
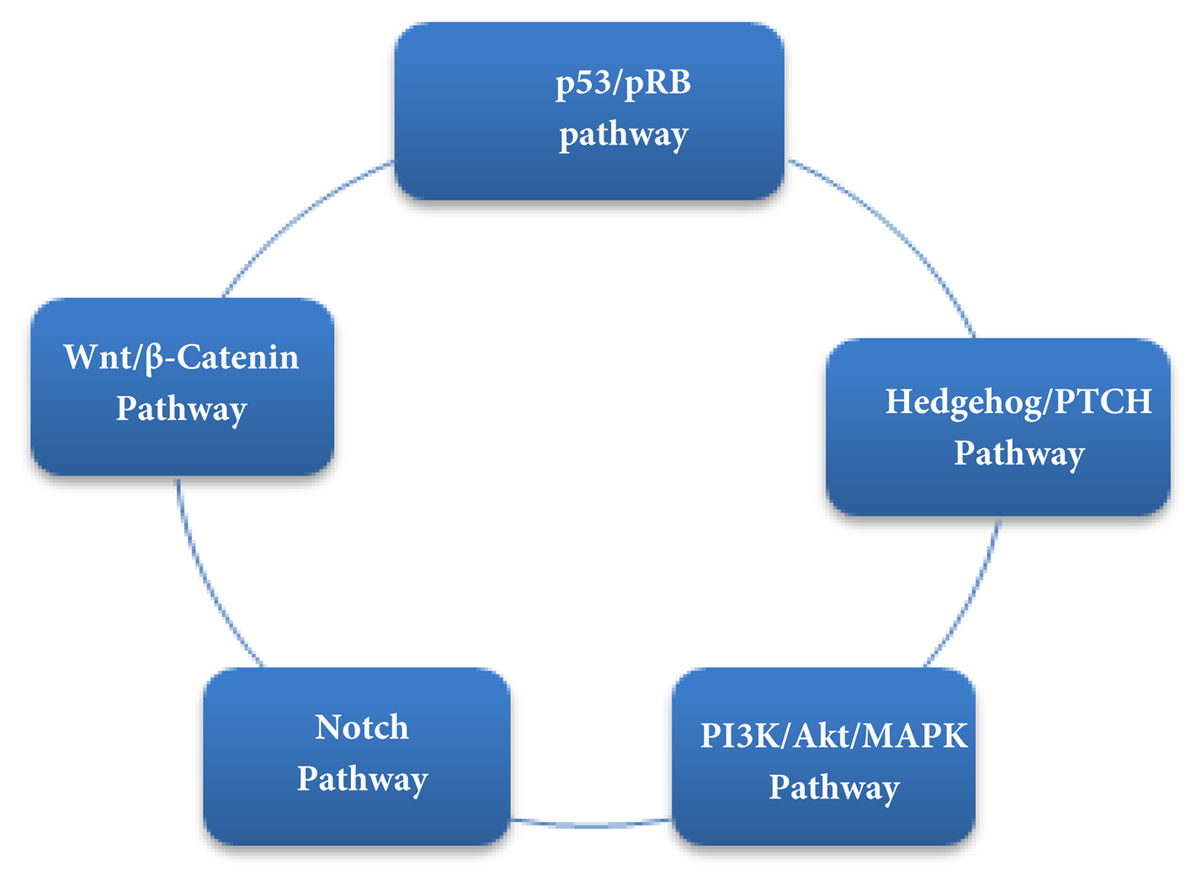
Signaling pathways involving meningiomas. These are the main pathways that have been linked to meningioma proliferation, malignancy progression, and tumorgenesis. The p53/pRB pathway plays a major role in the cell cycle by controlling the transition from G1 to S phase through the tumor suppressor gene pRB. The hedgehog/PTCH pathway has genes that are responsible for cell growth activation and suppression regulated by the SMO gene but when activated GLI transcription factors can also be activated. The Notch pathway is mediated by a number of transmembrane and serves as an intracellular communication system relaying messages throughout the cell. The PI3K/AKT/MAPK are two pathways that have been linked to malignant menigiomas with high levels of phosphorylatedAkt found in Grade II/III menigiomas (PI3K/Akt) and recurrence rate of meningiomas increase with reduced levels of MAPK. The Wnt/*β* catenin pathway deals more with benign (Grade I) menigiomas than with higher-grade menigiomas because many benign menigiomas have shown deletions in the APC gene whichis a major gene of this pathway. (Choy, Kim, Nagasawa, et al. 2011).

Numerous pathways have been linked to meningioma proliferation and progression [4], which get activated by the many genetic factors that lead to these tumors (Figure 2). The pRB/p53 pathway has been linked to meningioma progression and grade (anaplastic meningioma). Mutations and other deletions of the p16 (INK4a), p15 (INK4b), and p14 (ARF), which all act as modulators in the pathway, keeps pRB from binding and inhibiting E2F transcription factors. This in turn keeps pRB from inhibiting cell cycle progression at the G1/S-phase checkpoint causing dysregulation in the cell cycle [37]. The p53 gene is a tumor suppressor that can induce cell cycle arrest, repair DNA, induce apoptosis, and act as a feedback inhibitor of the pRB pathway. The hedgehog (Hh) pathway is a conservative pathway that has genes that are involved in cell growth, proliferation, angiogenesis, matrix remodeling, and stem cell homeostasis.

Hh binds to PTCH suppressing the PTCH-mediated tonic inhibitor in the Smoothened (SMO) protein, a transmembrane protein. If SMO is activated, a series of cellular signals are implemented, culminating in the activation of GLI transcription factors like GLI1 (growth activator), GLI2 (growth activator), and GLI3 (growth repressor). The Notch pathway is mainly an intracellular communication pathway that is mediated by the transmembrane proteins Notch 1–4 [38–39]. HES1 expression is found in many meningiomas of all grades and has been linked to the increased expression of the Jagged ligand, Notch 1, and Notch 2. TLE2/TLE3 are enhancers that increase the activity of the Split family (corepressors), which modulates HES1. These enhancers have been linked to malignancy and aggressive meningiomas because they were upregulated in malignant meningiomas [39].

The PI3K/Akt and MAPK (phosphatidylinositol 3-kinase/mitogen-activated protein kinase) are two complex pathways activated in many meningiomas [10,40–43]. These pathways have significant functions in cell differentiation, growth, and apoptosis while mediating the actions of many meningioma growth factors. Activating PI3K leads to AKT phosphorylation and p70 (S6K) activation by way of rapamycin (mTOR), mammalian target and regulator of multiple cancer cell processes. High levels of phosphorylated AKT are found of in Grade II (atypical) and Grade III (anaplastic) meningiomas but not in benign meningiomas. Activation of MAPK pathway upstream has been linked to Ras activation that phosphorylates Raf and MAPK. Reduced MAPK activation leads to a higher recurrence of meningioma while other pathways are linked to meningioma proliferation and progression like the PI3K/AKT pathway [39].

WNT/Beta-Cantenin is another pathway associated with meningiomas with mutations of two genes linked to this pathway: APC (adenomatous polyposis coli) and E-cadherin. Benign meningiomas have been shown to have deletions or other losses in the APC gene, a tumor suppressor, but these changes have not been documented in higher-grade tumors [44,45]. Losses of E-cadherin function have been found in one-third of all meningiomas and increased translocation of beta-cantenin into the nucleus has also been found in malignant meningioma. The APC tumor suppressor is an important modulator of the WNT/Beta-Cantenin pathway and the presence of E-cadherin decreases invasiveness and recurrence of meningioma [45].

Growth factors have also been linked to meningiomas through their overexpression and transformation. PDGF-BB (platelet-derived growth factor BB), with its receptor PDGFR-Beta, is often overexpressed in Grade II and Grade III meningiomas but not so much in benign meningiomas [13]. By activating the PI3K/Akt/MAPK pathways, PDGF-BB mediates its growth regulation. Anti-PDGF-BB agents can inhibit meningioma growth and proliferation. Epidermal growth factor receptor (EGFR) can be activated through an autocrine loop by expression of EGFR ligands, EGF, and TGF-alpha (transforming growth factor alpha; increased expression linked to aggressive meningioma proliferation). Other noted growth factors and receptors found in menigiomas are: TGF-Beta and its receptors, SDF1 and receptor CXCR, BMP (bone morphogenic protein; forms another autocrine loop that has been linked to meningioma proliferation) and BMPR, IGF, HER2, somatostatin, fibroblast growth factor, and placental growth factor, and Cox-2 [13].

## Current Challenges and Treatments in Meningiomas

For the majority of patients with benign meningiomas and a subset of patients with atypical meningioma standard therapy with surgery and radiation are effective in achieving tumor control. However, in higher-grade tumors that recur following surgery and radiation therapy current treatment options for these patients are currently inadequate and pose a therapeutic challenge. To date chemotherapy has had only a very limited role in the treatment of meningioma. There have been reports of small numbers of patients with malignant meningiomas who responded to recombinant interferon *α*-2b, hydroxyurea, temozolomide (Temodar, Schering- Plough; TMZ), and topoisomerase inhibitor irinotecan (Camptosar, Pfizer; CPT-11), but the evidence is mostly anecdotal. In a retrospective reviewof 14 patients with recurrent or progressive meningioma were treated with bevacizumab for 6 months and a progression free survival rate of 86% was reported after the follow-up examinations. [28,48].

Establishment of an effective therapeutic regimen for recurrent or progressive meningiomas is hampered by the relative lack of understanding of the molecular pathogenesis of meningiomas. Secondly, overexpression of various growth factors in meningiomas including PDGF, EGF, and VEGF and their receptors, and signal transduction pathways such as the Ras/MAPK, PI3K-Akt, and PLC- *γ*1-PKC pathways have been implicated, but their relative significance is largely unknown. As a result, the critical molecular targets remain to be elucidated. Another factor limiting progress in the development of more effective therapies for meningiomas is the lack of preclinical animal models. The lack of data regarding the natural history of untreated meningiomas is another important limiting factor that impedes progress. Without such data it is difficult to know if the periods of disease stabilization reported in various studies represent a response to therapy or the natural course of the disease. The current standard of care for grade I meningioma is surgical resection followed by imaging surveillance. In the case of atypical meningioma, radiation is often recommended, especially in cases with significant residual disease on post-operative imaging. In anaplastic meningioma (grade III), radiation is always recommended.

## Potential Treatments for Meningiomas

A better understanding of the molecular mechanisms including aberrations in cell signaling involved in meningioma pathogenesis may not only lead to the identification of novel diagnostic and prognostic marker but will also facilitate the development of new pathogenesis-based therapeutic strategies. Relatively little work has been conducted in understanding the growth factors, their receptors, and the signal transduction pathways that are critical to meningioma growth. Platelet-derived growth factor (PDGF), EGF, VEGF, IGF, TGF- *β*, and their receptor tyrosine kinases, together with their downstream signaling pathways including the Ras/MAPK pathway, the PI3K/Akt pathway, the PLC-*γ*1-PKC pathway, and the TGF-*β*-SMAD pathways, are all thought to be important in meningioma growth. Other potentially attractive therapeutic targets in meningioma include IGFR-2, histone deacetylase, NF*κ*B, HSP90, JAK/STAT, checkpoint kinase, and possibly Src kinase,focal adhesion kinase, and hypoxia-inducible factor 1*α*.

Gene-centered treatments that cater to the tumor genome and molecular profile of meningioma can also be another treatment paradigm for meningioma in the future. Gene silencing is one example of a potential therapy that can be effective against menigioma because this treatment will correct the alteration to genes causing meningioma proliferation. Another idea is to regulate major factors that can lead to meningioma proliferation like increased levels of YAP in the brain. In an experiment done on meningiomas, si-RNA was utilized to reduce the levels of YAP in cells that lack merlin due to NF2 mutations [7]. Finding supplements or replacements for genetic factors that are lost due to genetic aberrations is another potential idea for treatment when the gene itself can’t be fixed or silenced. Inhibiting meningioma angiogenesis is another major topic to address in future meningioma treatment paradigms. Menigiomas are vascular tumors VEGF-A and its receptor, VEGFR-1, regulate the development of peritumoral edema in tumors as well as neovascularity. Gliomas are most frequently associated with peritumoral edema; however, meningiomas have shown high variability in causing this condition [29]. 84% of menigiomas express VEGF while 67% express VEGFR [4]. Inhibiting VEGF and VEGFR may lead to decreased meningioma angiogenesis and some therapeutic drugs that have helped in this includes Sorafenib, Sunitinib, and the anti-VEGF antibody, bevacizumab. Since PDGF is a known meningioma growth factor, a receptor antagonist would be a viable treatment option and good direction to take future meningioma treatments. Additionally, a somatostatin antagonist could also be an effective treatment against meningioma proliferation in the CNS.

Deficient apoptotic mechanisms in cells also must be addressed when discussing future meningioma treatments. There are two main apoptotic pathways: an intrinsic and extrinsic pathway. The intrinsic pathway is regulated by the Bcl-2 protein family, depolarizes the mitochondrial membrane, and activates caspase 9 followed by caspase 3. Overexpression of either Bcl-2 or Bcl-XL has made tumor cells resistant to stimuli that induce apoptosis (cytotoxic agents, etc.). The extrinsic pathway works by activating cell death receptors through the cleavage of caspase 8 and is stimulated when receptors on the surface of a cell are activated by TRAIL, *TNF-related apoptosis-inducing ligand*. Molecules such as ABT-737, anti-sense Bcl-2, and mimetic peptides (BH3) along with IAPs (endogenous apoptosis suppressors) and synthetic retinoids (fenretinide, etc.) are all under consideration as possible ways to induce apoptosis in meningioma cells. Two other areas of interest for future treatments of meningioma are cell cycle inhibition and invasive metastasis inhibition. By inhibiting the activity of cyclin-dependent kinase (CDK) activity through the use of CDK inhibitors and endogenous CDK inhibitor complexes, cell cycle activity may be controlled for meningioma cells slowing proliferation. Furthermore, oral agents that can be used for long-term dosing together with other CDK inhibitors, targeting agents, or cytotoxic agents as a combination treatment may be more effective than activity of single-agents. Invasive meningiomas have shown over expression of metalloproteinase (MMP-2, MMP-9, SPARC, tenascin, Stremelsin-3) while TFPI-2 is over expressed in malignant meningiomas. TFPI-2 (tissue factor pathway inhibitor-2) secreted by all vascular cells due to the fact it’s an extracellular matrix-associated Kunitz-type serine proteinase inhibitor. Meningioma, being highly vascular in nature, secretes high levels of this proteinase, which has been linked to meningioma angiogenesis, metastasis, and invasion. Cilengitide (*α*V*β*3 and *α*Vv*β*5 intergin antagonist;) has shown some action against these two factors that has been linked to tumor angiogenesis and invasion in clinical trials as a glioma treatment and may also be effective against meningioma ( V 3 and Vv 5 are expressed in menigiomas).

## Conclusion

Meningioma is the second most common primary intracranial tumor found in adult patients. Therapeutic options for recurrent/progressive meningiomas are limited. Progress in identifying alternative forms of therapy or novel therapies for these tumors has been hindered by poor understanding of the multiple genetic abnormalities, signaling pathways, and other yet unknown factors that influence their biological behavior. The development of meningioma cell lines, fresh meningioma slice cultures and animal models for study of tumor biology, screening of drugs and study of pharmacokinetics and pharmacodynamics is essential. This is especially necessary for meningioma because the relevant patient population is significant and so far limited resources have been devoted to the study of this tumor.

The major chromosomes that have been linked to menigiomas are chromosomes 1, 9, 10, 14, and 22 and these genes are some of the genes that are either altered or mutated. Furthermore, genes associated with chromosomes 2, 4, 7, 8, 11, 12, and 16 have also been linked to meningioma proliferation. Mutations of these genes lead to the activation of many signaling pathways that cause meningiomas to arise all over the CNS and different regions of the brain. (Pham, Zada, Mosich, et al. 2011).

## Figures and Tables

**Table 1:** Scale by which the World Health Organization (WHO) gradess meningiomas.

WHO	Grade Description
Grade I (Benign)	Any histologic pattern other than clear cell, chordoid, papillary, or rhabdoid. Lacks criteria of atypical or an aplasticmeningioma
Grade II (Atypical)	Mitoses: 4–19 (10 hpf) Macronuclei, spontaneous necrosis, hypercellularity, small cell formation, sheeting architecture (Any 3 of these 5 parameters or possibly more) Meningioma protrudes into the parenchyma of the brain Clear cell or chordoid cell types
Grade III (Anaplastic)	Mitoses: 20+ (10 hpf) Apparent anaplasia (carcinoma/sarcoma-like histology) Rhabdoid or papillary cell type

**Table 2:** Genes associated with meningiomas.

Gene (Location)	Genetic Product/Function
NF2 (22q12.2)	Merlin (Protein); Tumor Suppressor
c-sis (22q13.1)	B-chain (PDGF-B); Growth Factor
BCR (22q11)	bcr (protein); GTPase activator, serine/threoninekinase (Wnt pathway)
TP73 (1p36.3)	p73; Apoptosis, Blocks proapoptotic function
PTEN (10p23.3)	phosphatidylinositol-3, 4,5- trisphosphate 3-phosphate; Tumor Suppressor
SUFU (10q24)	sufu (protein); Negative regulator in hedgehog pathway
sufu (protein);	Negative regulator in hedgehog pathway
AKT1 (14q32.32)	Protein Kinase B; Serine/Threonine Kinase
c-fos (14q24.3)	c-fos (protein); Transcription Factor
CDNK2A(9p21)	p16; Tumor Suppressor, Cell Cycle Progression (G1/S phase)
CDNK2B(9p21)	p15; Tumor Suppressor, Cell Cycle Progression (G1/S phase)
ARF (9p21)	p14; Tumor Suppressor, Cell Apoptosis Regulation
PTCH1 (9q22.3)	patched protein; Hedgehog Pathway Receptor
c-myc (8q24)	c-myc (protein); Transcription Factor
c-mos (8q11)	c-mos (protein); Serine Kinase
SFRP1 (8p11.21)	secreted frizzled-related protein 1; Extracellular signaling ligand secretion (Wnt pathway)
Ha-ras (11p15.5)	p21; Cyclin-dependent kinase inhibitor
IGF2 (11p15.5)	insulin-like growth factor 2; Hormone
bcl-2 (18q21.33)	bcl-2 (protein); Apoptosis regulator
STAT3 (17q21.2)	signal transducer & activator of transcription 3;Transcription factor
SMO (7q32.1)	smoothened GPCR protein; Cell Localization (Hedgehog Pathway)
GLI1 (12q13.3)	zinc finger proteins; Transcription Factor (Hedgehog Pathway)
FOXM1 (12p13.3)	forkhead box protein M1; Transcription Factor (Hedgehog Pathway)
GLI2 (2q14.2)	zinc finger proteins; Transcription Factor (Hedgehog Pathway)
GLIS2 (16p13.3)	zinc finger proteins; Transcription Factor (Hedgehog Pathway)
CDH1 (16q22.1)	E-cadherin; Cell Adhesion (Wnt Pathway) sufu (protein);
